# Ultrastructural analysis of breast cancer patient-derived organoids

**DOI:** 10.1186/s12935-021-02135-z

**Published:** 2021-08-10

**Authors:** Lorena Signati, Raffaele Allevi, Francesca Piccotti, Sara Albasini, Laura Villani, Marta Sevieri, Arianna Bonizzi, Fabio Corsi, Serena Mazzucchelli

**Affiliations:** 1 Dipartimento di Scienze Biomediche e cliniche “L. Sacco”, Università di Milano, 20157 Milan, Italy; 2 Istituti Clinici Scientifici Maugeri IRCCS, 27100 Pavia, Italy

**Keywords:** Transmission electron microscopy, Patient-derived organoids, Breast cancer

## Abstract

**Background:**

Breast cancer Patient Derived Organoids (PDO) have been demonstrated to be a reliable model to study cancer that promised to replace and reduce the use of animals in pre-clinical research. They displayed concordance with the tissue of origin, resuming its heterogenicity and representing a good platform to develop approaches of personalized medicines. Although obtain PDOs from mammary tumour, was a very challenging process, several ongoing studies evaluated them as a platform to study efficacy, sensitivity and specificity of new drugs and exploited them in personalized medicine. Despite tissue organization represented a crucial point to evaluate in a 3-dimensional model, since it could influence drug penetration, morphology of breast cancer PDOs has not been analysed yet. Here, we proposed a complete ultrastructural analysis of breast PDOs obtained from tumour and healthy tissues to evaluate how typical structures observed in mammary gland were resumed in this model.

**Methods:**

81 samples of mammary tissue (healthy or tumour) resulting from surgical resections have been processed to obtain PDO. The resulting PDOs embedded in matrigel drop have been processed for transmission electron microscopy and analysed. A comparison between ones from healthy and ones from cancerous tissue has been performed and PDOs derived from tumour tissue have been stratified according to their histological and molecular subtype.

**Result:**

The morphological analysis performed on 81 PDO revealed an organized structure rich in Golgi, secretion granules and mitochondria, which was typical of cells with a strong secretory activity and active metabolism. The presence of desmosomes, inter and intracellular lumens and of microvilli and interdigitations signified a precise tissue-organization. Each PDO has been classified based on whether or not it possessed (i) peripheral ridges in mitochondria, (ii) intracellular lumens, (iii) intercellular lumens, (iv) micro-vesicles, (v) open desmosomes, (vi) cell debris, (vii) polylobed nuclei, (viii) lysosomes and (ix) secretion granules, in order to identify features coupled with the cancerous state or with a specific histological or molecular subtype.

**Conclusion:**

Here we have demonstrated the suitability of breast cancer PDO as 3-dimensional model of mammary tissue. Besides, some structural features characterizing cancerous PDO have been observed, identifying the presence of distinctive traits.

**Supplementary Information:**

The online version contains supplementary material available at 10.1186/s12935-021-02135-z.

## Background

During the last decade, the employment of organoids has allowed to realize in vitro 3-dimensional structures able to reproduce features of the original tissue. Recently, organoids of different organs have been produced either using primary cells obtained from surgical resections (i.e. patient-derived organoids, PDO), either using embryonal or pluripotent stem cells [1, 2], resulting in the publication of *in vitro* methods well described and reproducible [3–5]. Indeed, the study of these in vitro 3-dimensional models, and in particular the exploitation of PDO could help the exploration of complex molecular mechanisms that drive disease progression. They could also have a role in regenerative therapy with the opportunity to transplant in vivo organoids produced in the laboratory [6]. Moreover, the use of PDOs as model of disease could reduce the employment of animal with a significant impact in 3Rs principles. PDOs could be also treated as a cell line, so they could be amplified and stored in liquid nitrogen allowing the creation of organoids biobanks [7, 8].

Here, we focused our attention on breast cancer (BC) PDOs. Indeed, BC is a challenging cancer type, representing a major cause of cancer-related death in the female worldwide population [9]. Therefore, the opportunity to better study in vitro BC response to different therapies and to develop tailored treatment strategies was an intriguing option. A BC organoid biobank has been created, exploring the possibilities opened by PDO in drug screening. Indeed, several studies have shown that the efficacy, sensitivity and specificity of drugs tested on PDO, compared to two-dimensional cultures, were closer to the in vivo condition [8, 10]. Moreover, also the association between the BC genotype and the in vitro anticancer drugs response on BC organoids, could help in define genetic determinants of cancer resistance. On the other hand, also their employment in the study of tumorigenesis represented a very interesting application, thanks to the advent of CRISPR/Cas9 technology. Recently, BC organoids have been edited by this method to investigate the driver genes involved in the tumorigenesis process [11, 12].

Otherwise PDOs morphology has never been analysed in depth, despite tissue organization represented a crucial point to evaluate, since it had an emerging role in drug’s penetration. Of date, ultrastructural characterization of BC tissue by transmission electron microscopy is very poor in literature, being referred to small case studies [13–16]. In detail, mammary normal tissue has been characterized by easily identifiable myoepithelial cells that surrounded the lobule and were in close proximity to basement membrane, which was fragmented and often absent in cancerous ones. Mammary epithelial cells appeared cubic or prismatic, and were characterized by few cytoplasmic organelles and by microvilli protruding into the lumen of the lobule. Cells were joined by desmosomes, while the nuclei appeared invaginated or polylobed and mainly constituted by heterochromatin. In the tumour tissue, cells were rich of filaments and of intracellular lumens, which were features also shared with healthy controls, although more uncommon [17]. Despite this, a better knowledge about ultrastructure of this in vitro 3D model could open novel scenarios about the nature of BC tissue and hence on the subcellular effects of different kind of treatments. Here, we reported a complete ultrastructural analysis of PDOs obtained from healthy and tumour tissues that summarized morphological features of them.

## Materials and methods

### Sample collection

**Fig. 1:**
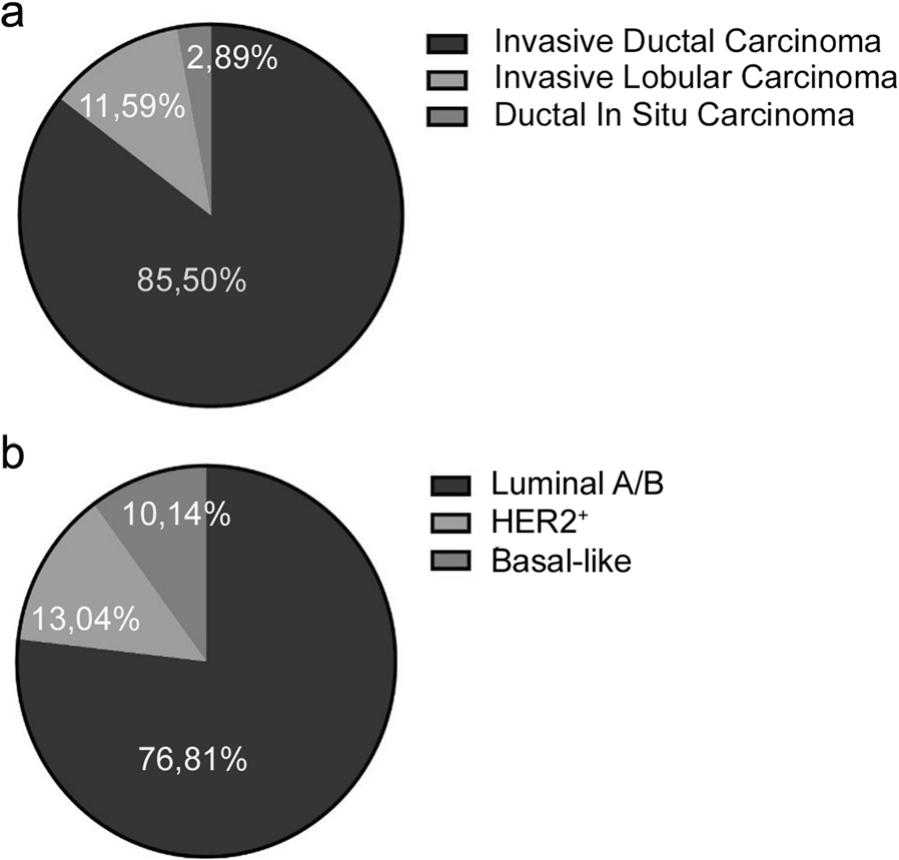
Molecular and histological distribution of tumour tissues collected for PDOs development. **a** Graphical representation of histological subtypes’ distribution of tumours used for PDO establishment. **b** Graphical representation of distribution of molecular subtypes of tumours used for PDO establishment

Eighty-one bioptic and surgical specimens have been collected from 74 consecutive, unselected patients treated at the Breast Unit of ICS Maugeri IRCCS (Pavia, Italy) from October 2018 to November 2020, after the signing an informed consent. Collection has been performed with the aim of storage samples in the “Bruno Boerci Oncological Biobank” of ICS Maugeri IRCCS. All surgical samples have been analysed by the pathologist, who has collected healthy and/or tumour tissue to be processed for PDO isolation, as previously described [18]. Healthy tissue represented normal mammary tissue collected from a mammary portion distant to cancer. Twelve were from healthy tissue, while 69 have been derived from tumour mammary tissue. Histological characterization of primary tumours revealed that 59 (85.50%) were invasive ductal carcinoma (IDC), while 2 (2.89%) were ductal in situ carcinoma (DCIS) and 8 (11.59%) were invasive lobular carcinoma (ILC) (Fig. 1a).

Distribution in each histological category reflected the frequency in which they appeared in the patient’s population and was due to a consecutive and not selected sampling. Immunohistochemistry assessment of receptors status evidenced that 9 patients (13.04%) were HER2-positive, while 53 (76.81%) were luminal A or B, and only 7 (10.14%) were basal-like breast cancer (Fig. 1b).

### PDO culture

Tumour and healthy samples from surgical resections and tumour from biopsies have been collected and stored at 4 °C in D-BSA (DMEM/High glucose supplemented with 1% Penicillin/Streptomicin and 10% Bovine serum albumin, BSA) until processing within 48 h. The tissue was transferred into a 10 cm Petri dish, where the adipose component was removed and the sample was cut with scalpel into 1 mm × 1 mm fragments. The sample was enzymatically digested in 10 mL Ad-DF +++ medium (Hyclone DMEM-F/12 1:1 supplemented with 10 mM HEPES, 1% Penicillin/Streptomicin and 1% L-glutamine) supplemented with 500 µL of Collagenase 20 mg/mL and 10 µL of Y27632 10 mM for 1–2 h at 37 °C. Undigested tissue was removed by filtering on sterile 100 µm cell strainer and PDOs were obtained by centrifuging the flow-through sample 5 min at 500 × g, 8 °C. Then, the supernatant was carefully removed and, the pellet was treated with 1 mL of Tac buffer for 10 min at 37 °C, if it contained red blood cells. PDOs were resuspended in the appropriate amount of Matrigel 90% (Corning, Matrix Basement Membrane Growth Factor Reduced Phenol Red Free, #356231) or BME (Bio-techne, Cultrex PathClear Reduced Growth Factor BME, #3533-010-02) and they were seeded in a pre-warmed multiwell plate. Once PDO-Matrigel drops were solidified, the culture medium (CM) was gently added (Table 1) and the plate was transferred into incubator for culturing. To ensure the best culture conditions, it is necessary to change medium thrice a week and to process BC organoids every 7-10 days by embedding or shearing in order to replace Matrigel/BME. The embedding procedure should be performed when the density of Matrigel/BME drop is too high and it is necessary to expand the sample, or in the case of the Matrigel/BME is dissolved or degraded. Instead, the shearing procedure should be performed when organoids were bigger than 100 µm.

**Table 1:** Composition of culture medium (CM) used for BC PDOs growth

Reagents	Supplier	Catalogue number	Final Concentration
DMEM/F12	HyClone	D8437	1X
L-glutamine	Corning	25-005-CI	1%
Penicillin/Streptomicin	Corning	30-003_CI	1%
Hepes	Sigma	83264	10 mM
Noggin conditioned medium	Home made	–	25×
B 27 supplement	Gibco	17504-044	1×
N-acetyl-cysteine	Sigma	A9165	1.25 mM
Nicotinamide	Sigma	N0636	0.2 mM
A 83-01	Tocris	2939	500 nM
Y-27632	ForLab	M1817	5 µM
R-spondin1 conditioned medium	Home made	–	10%
Primocin	Invivogen	ANT-PM-2	50 µg/mL
Human EGF	Peprotech	AF-100-15	5 ng/mL
FGF-10 HUMAN recombinant	Peprotech	100-26-25	20 ng/mL
KGF/FGF-7 Human recombinant	Peprotech	100-19-10	5 ng/mL
Heregulin-beta-1 Human recombinant	Peprotech	100-03-50	37.5 ng/mL
SB 202190	Sigma	S7067	500 nM

### Transmission electron microscopy

PDOs cultured for 2–4 weeks or surgical tissue were fixed for 2 h at 4 °C in cacodylate buffered 2.5% glutaraldehyde, postfixed in 1.5% osmium tetroxide dissolved in cacodylate buffer, dehydrated in ascending scale of ethyl alcohol and included in Epon. The ultrafine slices were stained with uranyl acetate and lead citrate and analysed by Tecnai Spirit Biotween electron microscope (FEI). Before to start ultrastructural characterization of BC-PDOs, all the collected samples have been preliminary observed and described in order to identify morphological categories useful for analysis. Morphological features identified during preliminary observations and then used for classification were the presence of: (i) peripheral ridges in mitochondria, (ii) intracellular lumens, (iii) intercellular lumens, (iv) micro-vesicles, (v) open desmosomes, (vi) cell debris, (vii) polylobed nuclei, (viii) lysosomes and (ix) secretion granules. At least 3 PDO with dimensions ranging from 70 to 150 μm have been evaluated for each sample. The whole PDO has been considered, examining about 100–200 cells/PDO and identifying whether a certain PDOs has fixed characteristic or not. A morphological feature has been attributed to a certain PDO only when it has been found in at least 75% of considered cells  (see Additional file 1). Intracellular lumens, intercellular lumens, micro-vesicles, polylobed nuclei and secretion granules has been studied at 2000–2500 magnification, while peripheral ridges in mitochondria, open desmosomes and lysosomes has been observed at 6000 magnification. Microvescicles have been observed at 20,000 magnification. Only in the last round, obtained results have been analysed, stratifying evaluated samples into tumour or healthy tissues and in histological and molecular subclasses.

### Statistical analysis

Variables were categorical, they were reported in tables as absolute numbers and percentages. Categorical variables were compared using χ2 test or Fisher exact test (choice based on the sample size in each category): Fisher exact test was conducted for each table in which even just one category had a sample size of n ≤ 5, while in the other cases a χ2 test was implemented Statistical significance was set at p < 0.05 (two tailed). Data analysis was performed using SAS software (v. 9.4, SAS Institute Inc., Cary, USA).

## Results

### PDO ultrastructure recapitulates mammary gland morphology

To evaluate reliability of PDO as a structural model of mammary gland, 81 PDOs obtained from not selected and consecutive biopsies and surgical resection performed by the Breast Unit of ICS Maugeri IRCCS (Pavia, Italy) from October 2018 to November 2020, have been selected for this study.

**Fig. 2:**
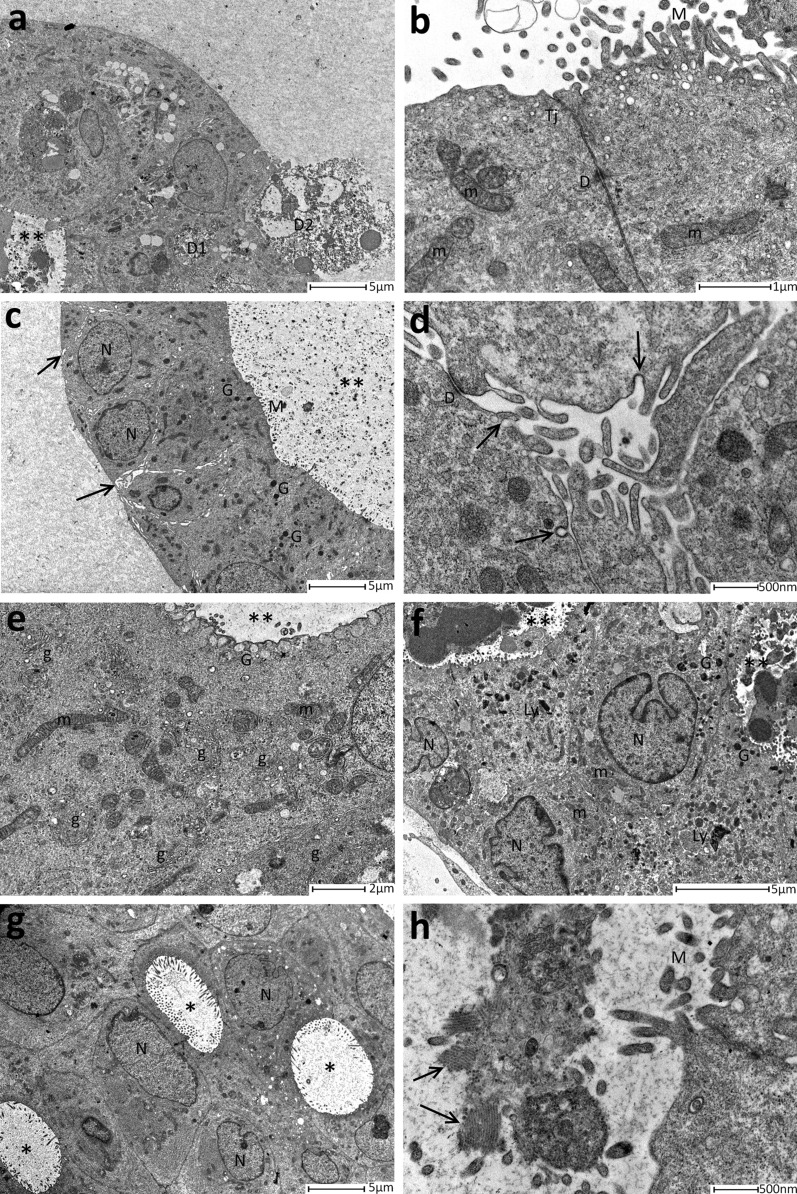
Features of PDOs derived from healthy and tumour mammary tissue. **a** Cellular debris could be detected both in intercellular lumens both outside of the organoid at small magnification. Neither basement membrane neither myoepithelial cells are present. **b** Tight junctions and desmosomes were visible between two cells that surrounded the lumen. **c** A large intercellular lumen with protein material inside was evidenced in this micrograph. In the basal layer of the cells, intercellular spaces (arrows) were dilated with interdigitation of the cytoplasmic membrane. Both basement membrane and myoepithelial cells were absent. **d** High magnification of the loosed intercellular spaces. The interdigitations between cells were evident, as pinocytosis vacuoles (arrows). **e** In this photomicrograph, the large number of Golgi complex was well represented. **f** Two intercellular lumens containing various material, lysosomes and protein granules were displayed in this micrograph. The nuclei appeared polylobed. **g** Representative image of numerous intracellular lumens with microvilli inside. **h** Representative micrograph of an internal lumen showing spherical and rod-shapes material (arrows). D1 = intercellular debris, D2 = outsider debris, **= intercellular lumens containing material. TJ = tight junction, D = desmosome, m = mitochondria, M = microvilli, N = nuclei, G = secretory granules, g = Golgi apparatus, Ly = lysosome. PDOs reported in this figure were derived from IDC Luminal A/B (**a**, **b**, **f**, **g**, **i**), IDC HER2+ (**d**), ILC luminal A (**e**, **h**) or from DCIS luminal A (**c**)

All the 81 organoids had spheroidal or ovoid shape and were constituted by cylindrical or cubic cells of about 10 μm (Fig. 2a), which were closely linked together by small desmosomes (Fig. 2b). PDO’s cells were organized in a polarized manner, where the apical secretory portion of the cell faces the interior of the organoid. Moreover, cells displayed numerous Golgi apparatuses mainly located in the perinuclear area, suggesting an intense activity in lipid and protein maturation (Fig. 2e). This feature, coupled with the presence of secretion granules in the apical part, represented a signal of a strong secretory activity (Fig. 2c, f). Also, many mitochondria have been detected in organoid’s cells, revealing an active metabolic activity (Fig. 2e). In the PDO structure, cell borders were dilated and occupied by cytoplasmic interdigitations, while there was no evidence of basement membrane or even of basement myoepithelial cells (Fig. 2d). All the observed PDOs displayed at least one intercellular lumen inside. Despite being present in every sample, these lumens were different in size, ranging from only one of considerable size, which included most of the organoid, to many small lumens irregular in shape (Fig. 2a–c, e–h). Amorphous secretory material and rarely cellular debris of various sizes were contained inside intercellular lumen in rounded or stick-shape microvescicles (Fig. 2a–d, f, g). Moreover, they were characterized by the presence of tight junctions and of eversions of cell membrane similar to microvilli (Fig. 2c, i). Also, intracellular lumen has been detected including also in this case microvescicular structures (Fig. 2g–i). The presence of all these features evidenced that PDO is a well-organized three dimensional entity, which reflected structural features also observed in mammary tissue (Fig. 2).

**Fig. 3:**
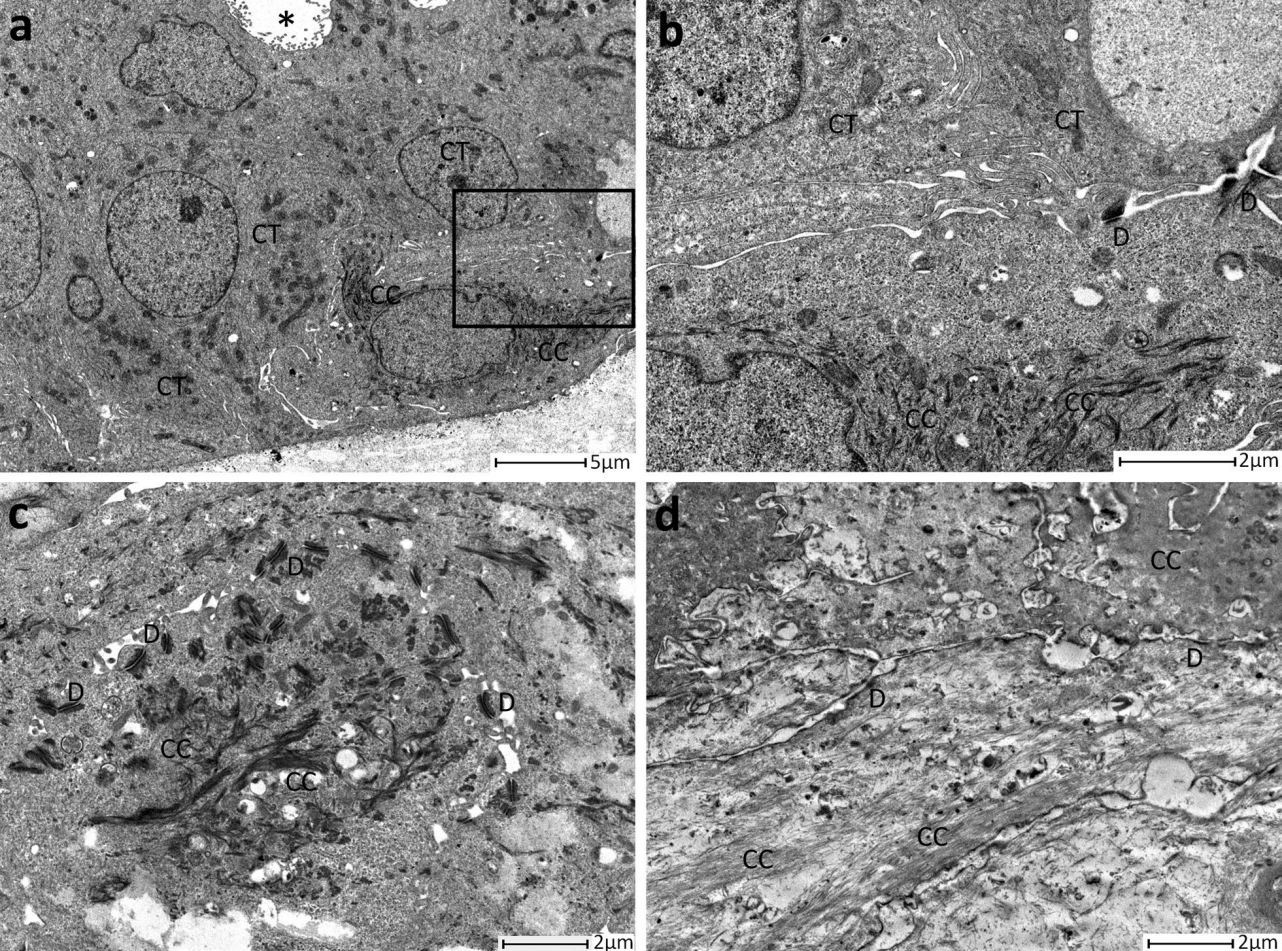
**a** Representative image of a single cell with numerous keratin (CC) filaments. This cell was in basal position and in closed contact with tumour cells (CT). **b** Magnification of panel **a** that depicted one cell in basal position, full of keratin (CC) filaments. Also, the neighbouring tumour cells (CT) were displayed. **c** Magnification of keratin (CC) filaments. **d** Representative image of keratinized cells. The cytoplasm is full of keratin filaments (CC), “ghost” desmosomes and cellular organelles were absent. Intercellular lumen = *, D = desmosome

Finally, in 9 (11.11%) PDOs samples, some keratin-rich cells have been detected (Fig. 3). These cells were spotted inside the organoid structure or were clusterized in large aggregates that sometimes involved the whole PDO. They displayed a cytoplasm rich in filamentous structures, that suggested the start of the keratinization process. Indeed, the enrichment in keratins expression and the presence of traces of keratinization were features observed in squamous cell carcinoma, which is a very rare and aggressive BC subtype and seemed to be related to a transition to metaplastic cell carcinoma [19–22].

### Analysis to identify morphological pattern of tumorigenicity: a comparison between PDO from healthy and cancerous tissue

**Fig. 4:**
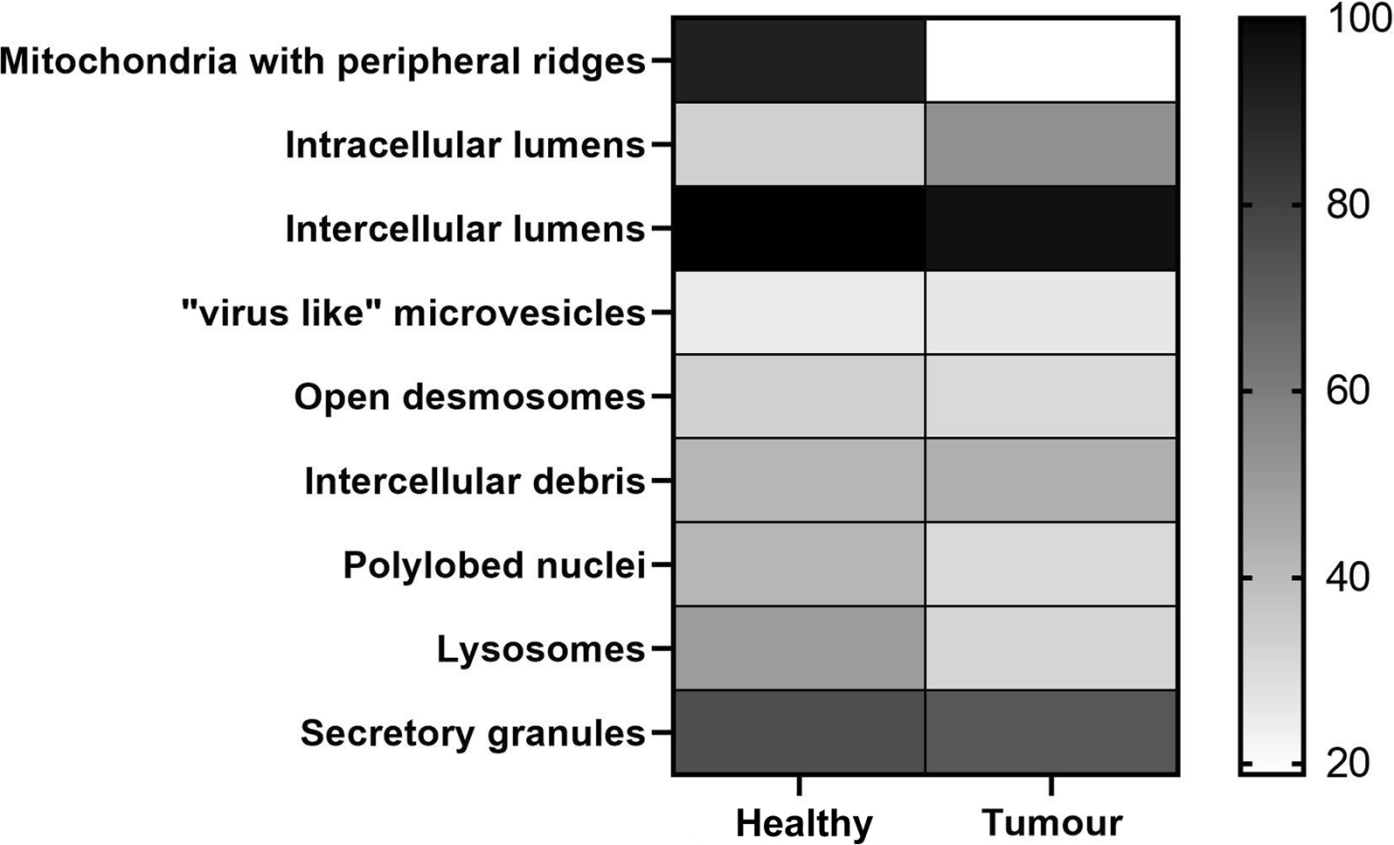
Heat map representation of morphological parameters evaluated by TEM of mammary PDO, stratified for localization of sampling

In our morphological analysis, organoid samples have been divided into 2 groups following the localization of tissue sampling as, healthy and tumour PDOs. They were analysed underlining several parameters, such as the presence of (i) peripheral ridges in mitochondria, (ii) intracellular lumens, (iii) intercellular lumens, (iv) micro-vesicles, (v) open desmosomes, (vi) cell debris, (vii) polylobed nuclei, (viii) lysosomes and (ix) secretion granules (Fig. 4; Table 2).

**Table 2:** Summary of frequencies in which these morphological parameters have been observed in mammary PDO, obtained from healthy and tumour tissue

	Healthy	Tumour	P value
Mitochondria with peripheral ridges			
Yes	11 (91.66%)	13 (18.84%)	0.01
No	1 (8.34%)	56 (81.16%)	
Intracellular lumens			
Yes	4 (33.33%)	37 (53.62%)	0.23
No	8 (66.67%)	32 (46.38%)	
Intercellular lumens			
Yes	12 (100%)	67 (97.10%)	1
No	0 (0%)	2 (2.90%)	
“Virus like” microvesicles			
Yes	3 (25%)	18 (26.08%)	1
No	9 (75%)	51 (73.92%)	
Open desmosomes			
Yes	4 (33.33%)	21 (30.43%)	1
No	8 (66.67%)	48 (69.57%)	
Intercellular debris			
Yes	5 (41.66%)	30 (43.47%)	1
No	7 (58.34%)	39 (56.53%)	
Polylobed nuclei			
Yes	5 (41.66%)	21 (30.43%)	0.51
No	7 (58.34%)	48 (69.57%)	
Lysosomes			
Yes	6 (50%)	22 (31.88%)	0.32
No	6 (50%)	47 (68.12%)	
Secretory granules			
Yes	9 (75%)	50 (72.46%)	1
No	3 (25%)	19 (27.54%)	

**Fig. 5:**
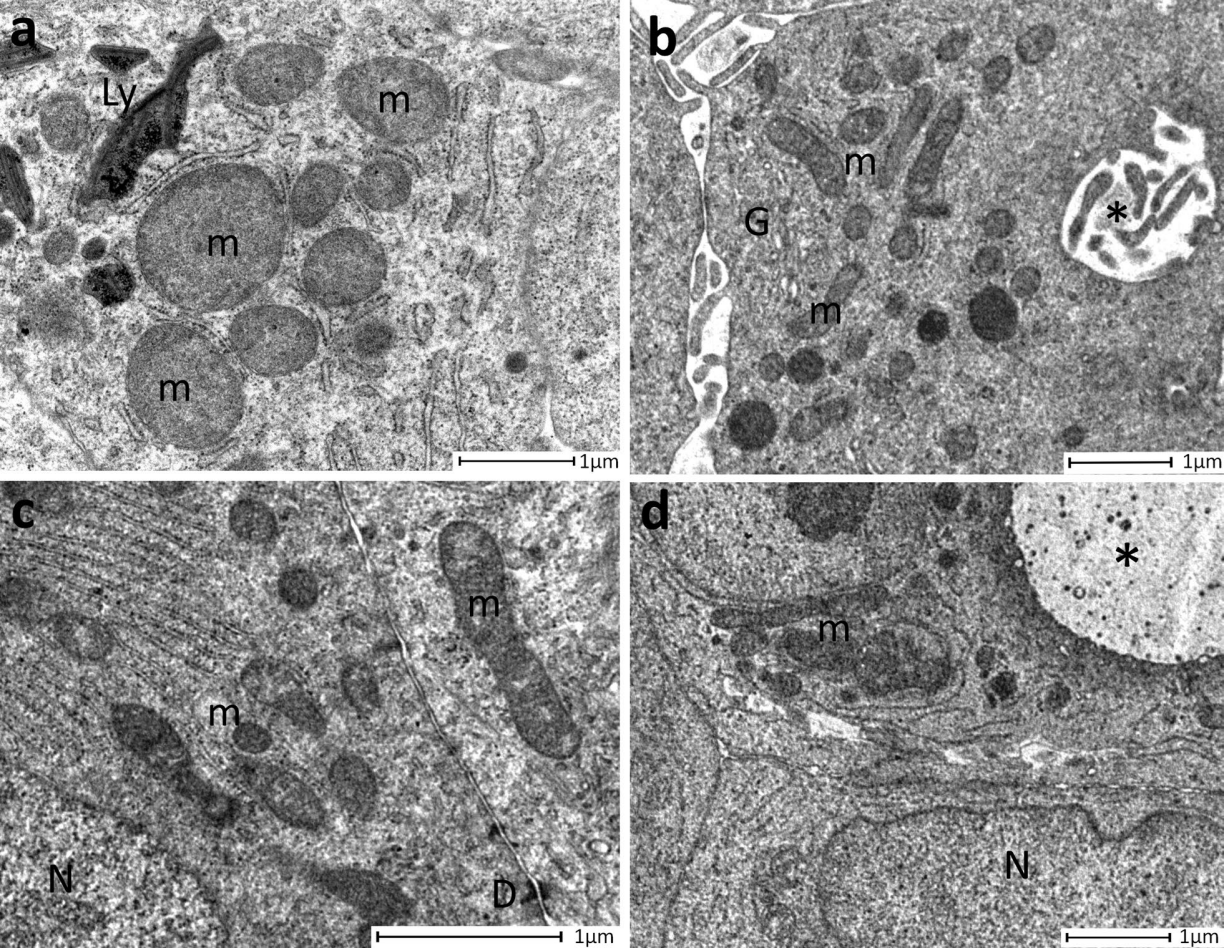
Analysis of mitochondria morphology. **a** Representative image of round mitochondria with few and peripherical ridges observed in PDO from healthy tissue. **b** PDO from invasive ductal carcinoma (IDC) evidenced long mitochondria with well represented ridges. **c** Elongated and rich-in ridges mitochondria observed in PDO derived from ductal in situ carcinoma (DCIS). **d** Representative image of elongated mitochondria identified in a PDO from invasive lobular carcinoma (ILC). M = mitochondria; Ly = lysosomes; G = Golgi apparatus; *= intracellular lumen; N = nucleus; D = desmosomes

The comparison between PDOs from tumour and healthy tissue highlighted that the former mostly displayed mitochondria elongated and rich in ridges (Figs. 4, 5; Table 2), while mitochondria from PDOs of healthy tissue were roundish, even of large dimensions, with few short ridges arranged peripherally.

**Fig. 6:**
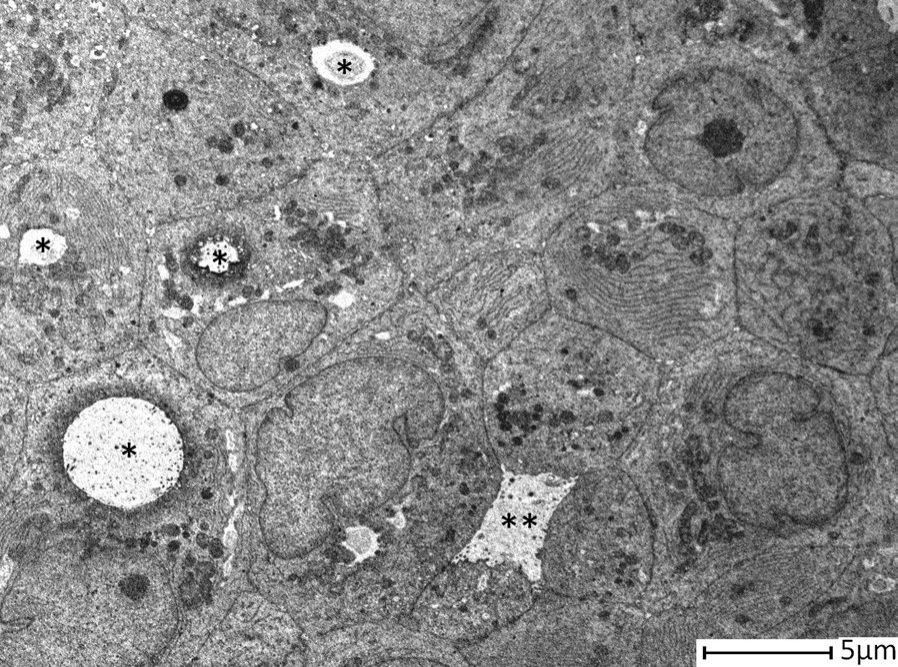
Representative image of intracellular lumens (*) of various sizes. A small intercellular lumen (**) was also highlighted

Although the presence of intercellular lumens was a feature detected in all assessed groups (Fig. 4, Table 2), the presence of intracellular lumens seemed to be a quality mostly attributable to PDO derived from tumour (Figs. 4, 6). Indeed, it has been detected only in 4 PDOs from healthy tissue (33.3%), while in tumour organoids this percentage reached 53.62% (37/69).

Moreover, comparing the frequencies of distribution of other structural features between organoids from healthy and tumour tissue, light differences have been detected in the presence of polylobed nuclei and of lysosomes, which were mostly represented in PDOs from healthy mammary tissue (41.66% vs 30.43% and 50% vs 31.88%, respectively) (Fig. 4; Table 2). Otherwise, distribution of microvesicles, desmosomes, intracellular debris and secretion granules were similar between PDOs from healthy and tumour tissue (Fig. 4; Table 2). In detail, closed desmosomes were few and mainly located in cells that bordered intercellular lumen, while open desmosomes, coupled with intercellular debris, were quite frequent (33.33% vs 30.43%, respectively in PDOs from healthy and tumour tissue) and suggestive of a marked cell turnover. Amorphous material, debris or “virus-like” microvesicles, both round and rod-shaped, could be found in the intracellular and intercellular lumens. Although the meaning of these formations was not clear, they probably were due to exosomes.

**Fig. 7:**
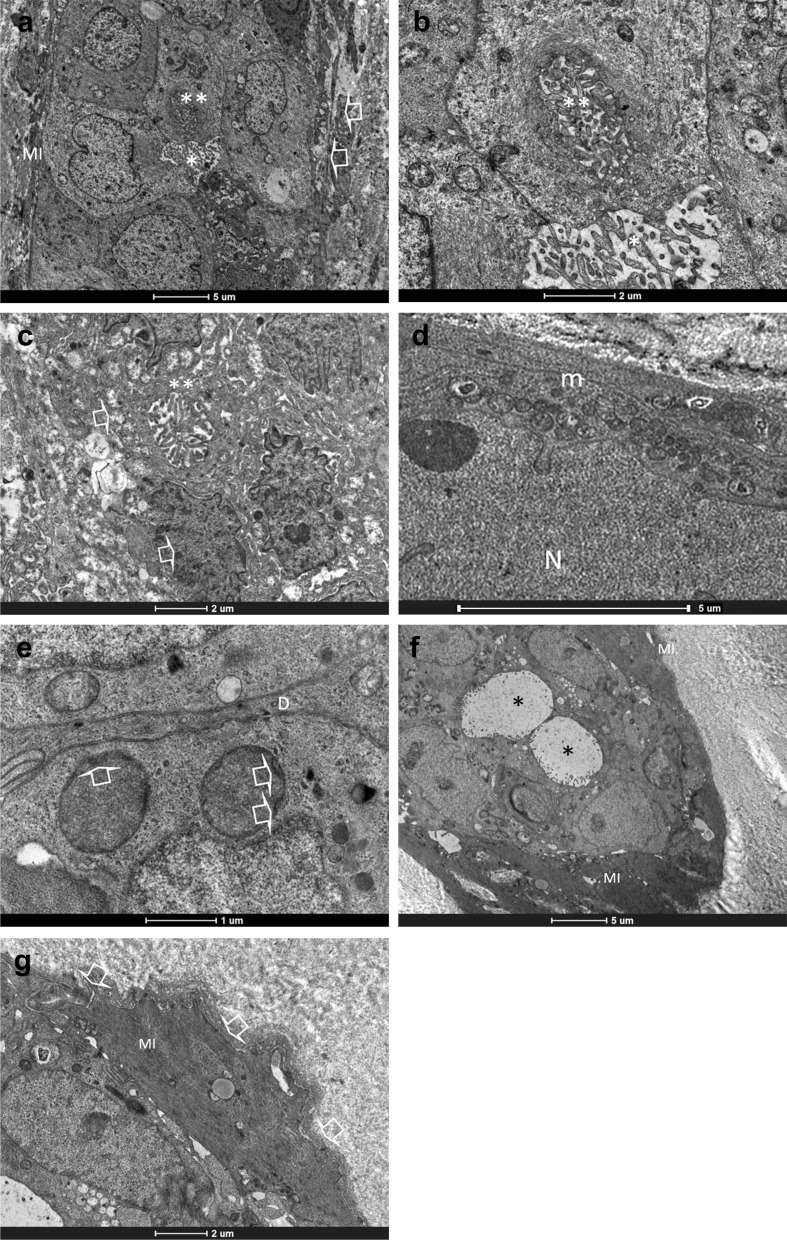
Transmission electron microscopy of healthy and tumour mammary gland. **a** Representative image of tumour mammary tissue. The lobule centrally displayed an intercellular lumen (*) and an intracellular lumen (**) with numerous microvilli inside them. Myoepitalial cells (MI) were discontinuous and basement membrane was absent (arrows). **b** Magnification of panel **a**, where the intracellular lumen (**) and the intercellular lumen (*) with their microvilli were clearly distinguishable. **c** Magnification of panel **a**, where could be detected an intracellular lumen (**) in the basal position. Neither myoepithelial cells, nor basement membrane could be recognized (arrows). **d** Representative image of mitochondria (m) rich in cristae from tumour mammary tissue. N= nuclei. **e** Representative image of healthy mammary gland, where two mitochondria with peripheral ridges (arrows) were clearly evident. D = desmosome. **f** Representative image of a lobule, where two intercellular lumens (*) were detecable, as well as myoepithelial cells (MI) that continuously surrounded the lobule. **g** Magnification of **f**. The basement membrane (arrows) and myopithelial cells (MI) were identifiable

To verify if features detected in mammary tumour-derived PDO, were found also in the tissue of origin, we have performed TEM analysis of tumour tissue. As already described in literature [14–17], in tumour mammary gland basement membrane was absent, as well as myoepithelial layer that sometimes appeared as discontinuous. Tumour cells were arranged to form some intercellular lumens of medium size, while numerous intracellular lumens were detected inside cells. In comparison to that observed in tumour mammary tissues, healthy ones displayed basement membrane and myoepithelial cells. Moreover, only intercellular lumens were detected, while intracellular lumens were absent (Fig.7). As observed in PDOs from healthy breast, mitochondria were characterized by few short peripheral ridges (Fig. 7).

**Fig. 8:**
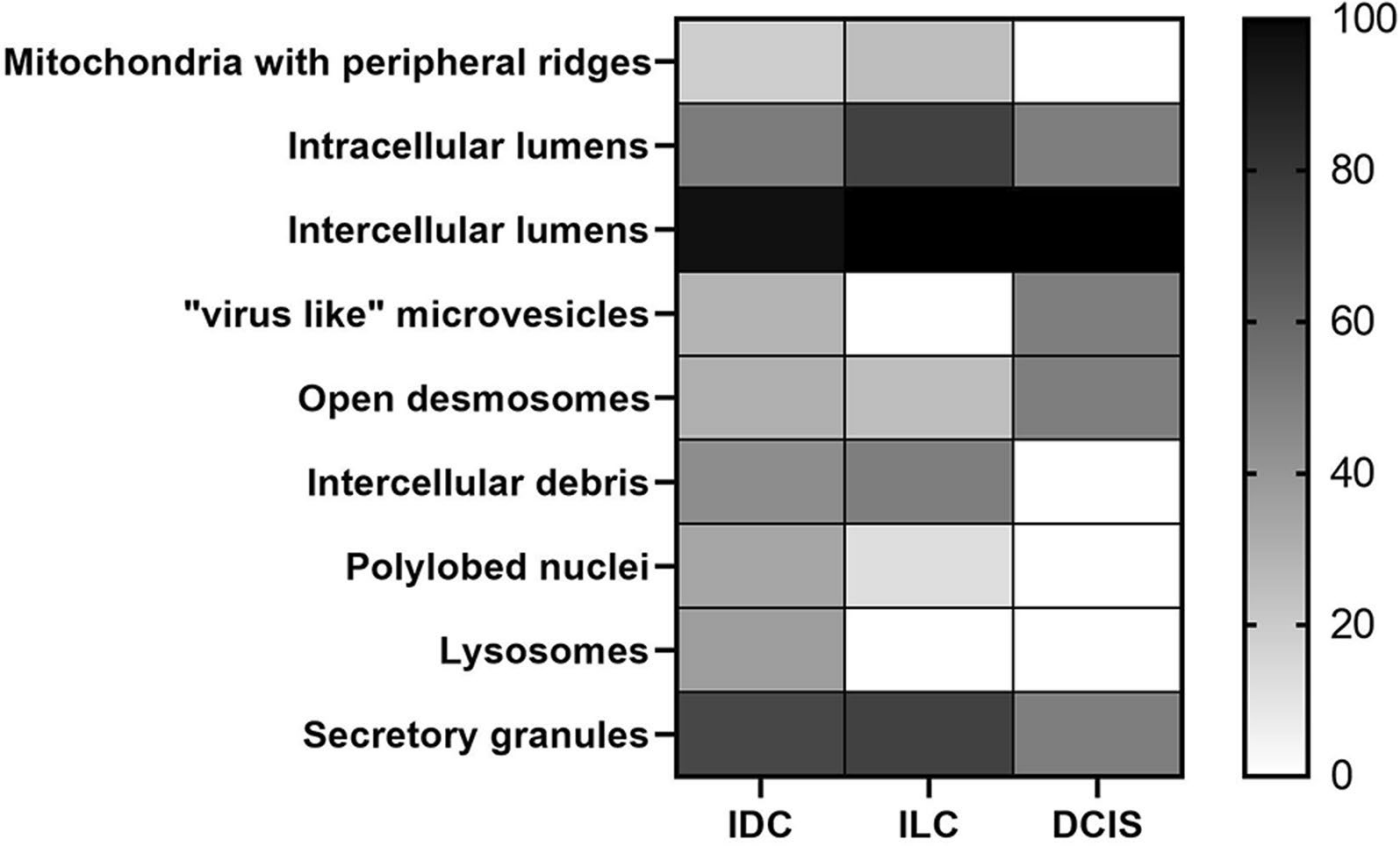
Heat map representation of morphological parameters evaluated by TEM of mammary PDO, stratified for histological subtype

Then, PDOs derived from tumour tissue have been stratified into 3 groups after histological characterization as, invasive ductal carcinoma (IDC), invasive lobular carcinoma (ILC) and ductal in situ carcinoma (DCIS). They were analysed as previously explained, underlining the structural and morphological parameters identified before (Fig. 8; Table 3).

**Table 3:** Summary of frequencies in which these morphological parameters have been observed in mammary PDO, obtained from different histological tumour subtypes

	IDC	ILC	DCIS
Mitochondria with peripheral ridges			
Yes	11 (18.64%)	2 (25%)	0 (0%)
No	48 (81.36%)	6 (75%)	2 (100%)
Intracellular lumens			
Yes	30 (50.84%)	6 (75%)	1 (50%)
No	29 (49.16%)	2 (25%)	1 (50%)
Intercellular lumens			
Yes	57 (96.61%)	8 (100%)	2 (100%)
No	2 (3.39%)	0 (0%)	0 (0%)
“Virus like” microvesicles			
Yes	17 (28.81%)	0 (0%)	1 (50%)
No	42 (71.19%)	8 (100%)	1 (50%)
Open desmosomes			
Yes	18 (30.51%)	2 (25%)	1 (50%)
No	41 (69.49%)	6 (75%)	1 (50%)
Intercellular debris			
Yes	26 (44.06%)	4 (50%)	0 (0%)
No	33 (55.94%)	4 (50%)	2 (100%)
Polylobed nuclei			
Yes	20 (33.89%)	1 (12.5%)	0 (0%)
No	39 (66.11%)	7 (87.5%)	2 (100%)
Lysosomes			
Yes	22 (37.28%)	0 (0%)	0 (0%)
No	37 (62.72%)	8 (100%)	2 (100%)
Secretory granules			
Yes	43 (72.88%)	6 (75%)	1 (50%)
No	16 (27.12%)	2 (25%)	1 (50%)

Results reported in Fig. 8 and summarized in Table 3 seemed to suggest different distribution of lysosomes and polylobed nuclei between PDOs from IDC and from ILC and DCIS, despite significant difference has been detected only between IDC and ILC (P < 0.05) in lysosomes. To date, microvesicles were not found in PDOs from ILC subtypes although this difference is not statistically significant.

Finally, PDOs from mammary tumour have also been stratified into 3 groups following molecular classification as, luminal A/B, Basal-like and HER2^+^. Also in this case, they were analysed, monitoring the presence of (i) peripheral ridges in mitochondria, (ii) intracellular lumens, (iii) intercellular lumens, (iv) micro-vesicles, (v) open desmosomes, (vi) cell debris, (vii) polylobed nuclei, (viii) lysosomes and (ix) secretion granules.

**Fig. 9:**
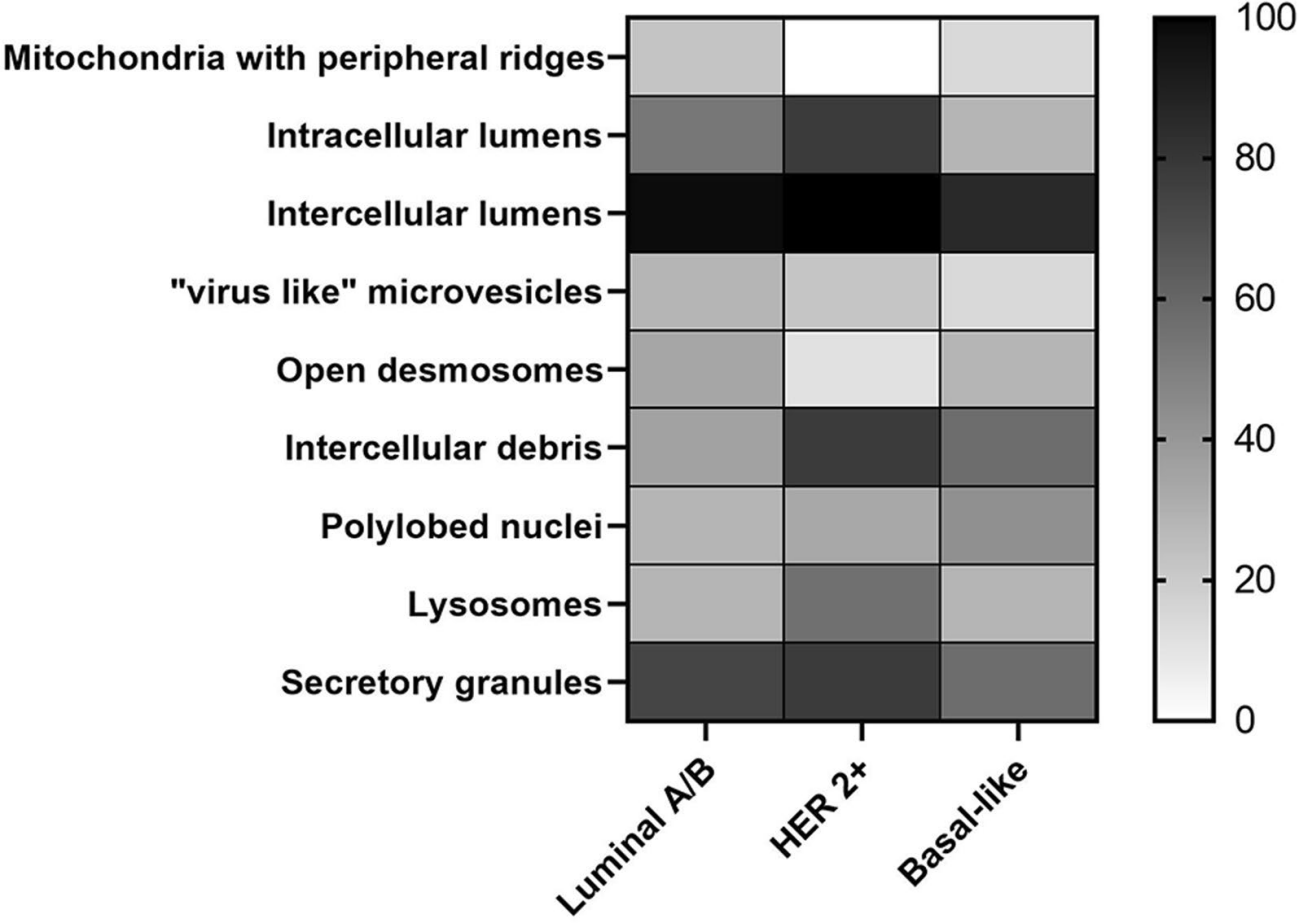
Heat map representation of morphological parameters evaluated by TEM of mammary PDO, stratified for molecular subtype

Results of our analysis have been summarized in Table 4 and in Fig. 9. The stratification of results following molecular subtype revealed that mitochondria with peripheral ridges were completely absent in PDO from HER2+ tumours (Fig. 9; Table 4). In the meantime, intercellular debris seemed to be characterizing features in this molecular subtype, as evidenced by the statistical significance (P < 0.02) obtained in comparison to luminal A/B subtype. PDO derived from basal-like tumours displayed ”virus-like” microvesicles and mitochondria with peripheral ridges only in one sample (14.28%), while all the other features (intracellular lumens, desmosomes, intercellular debris, lysosomes, secretion granules and polylobed nuclei) have been detected in a percentage of samples ranging from 20 to 60% (Fig. 9; Table 4). Luminal A/B-derived PDOs, as already observed for DCIS, were not distinguished from other molecular subtypes by the presence or by the absence of one of these morphological features (Fig. 9; Table 4).

**Table 4:** Summary of frequencies in which these morphological parameters have been observed in mammary PDO, stratified for molecular subtype

	Luminal A/B	HER 2+	Basal-like
Mitochondria with peripheral ridges			
Yes	12 (22.64%)	0 (0%)	1 (14.28%)
No	41 (77.36%)	9 (100%)	6 (85.72%)
Intracellular lumens			
Yes	28 (52.83%)	7 (77.77%)	2 (28.57%)
No	25 (47.17%)	2 (22.23%)	5 (71.43%)
Intercellular lumens			
Yes	52 (98.11%)	9 (100%)	6 (85.72%)
No	1 (1.89%)	0 (0%)	1 (14.28%)
“Virus like” microvesicles			
Yes	15 (28.30%)	2 (22.23%)	1 (14.28%)
No	38 (71.70%)	7 (77.77%)	6 (85.72%)
Open desmosomes			
Yes	18 (33.96%)	1 (11.11%)	2 (28.57%)
No	35 (66.04%)	8 (88.89%)	5 (71.43%)
Intercellular debris			
Yes	19 (35.84%)	7 (77.77%)	4 (57.14%)
No	34 (64.16%)	2 (22.23%)	3 (42.86%)
Polylobed nuclei			
Yes	15 (28.30%)	3 (33.33%)	3 (42.86%)
No	38 (71.70%)	6 (66.67%)	4 (57.14%)
Lysosomes			
Yes	15 (28.30%)	5 (55.55%)	2 (28.57%)
No	38 (71.70%)	4 (44.45%)	5 (71.43%)
Secretory granules			
Yes	39 (73.58%)	7 (77.77%)	4 (57.14%)
No	14 (26.42%)	2 (22.23%)	3 (42.86%)

## Discussion

Despite ultrastructural characterization of the breast tissue was very poor in literature [14, 16, 17, 23], this study on PDOs morphology evidenced that cell dimensions, shape and intercellular organization resembled to that previously observed in tissue (Fig. 7) and indicated a much more complex organization than 2D cultures. Moreover, the presence of intercellular lumens, tight junctions, numerous secretion granules and the formation of glandular hints with lumens rich in microvilli and secretion material evoked an attempt to re-build the structure of the lobule (Fig. 7) [14, 16, 17, 23]. The recovery of these typical features of mammary gland organization in PDO supported once more their reliability as model of BC prompting their suitability to study drug penetration. To date, PDOs seemed to lack of myoepithelial cells that surrounded the lobule and of the basement membrane, which were distinctive elements in normal mammary gland. Beside this, the invaginated or polylobed appearance of nuclei with the predominance of heterochromatin and the diffuse localization of intracellular lumen represented features of the breast [17]. Moreover, the presence of desmosomes and of numerous interdigitations suggested how PDO-forming cells worked synergistically. In PDO structure has also been identified a sort of “intercellular canaliculi”, which could have a role in cell nutrition and cell turnover, since they were full of pinocytosis vesicles and of cell debris. Overall, these data from 81 BC-PDOs described in depth ultrastructural features observed in this ex vivo three-dimensional model and corroborated its goodness for studies of drug penetration.

Despite the comparison between PDO from cancerous and healthy tissue evaluating the presence or not of some distinctive features has not allowed the identification of a morphological pattern of disease, it should be noticed that it has revealed significant differences in mitochondrial morphology. Indeed, PDO from healthy tissue displayed peripheral ridges, while those from tumours were rich in cristae. These differences were consistent to the high metabolic burden observed in cancer. Indeed, the high growth rate in cancers leaded to significant alteration in cell metabolism, since cancer cells needed to obtain energy from catabolic processes, but also should favour biosynthetic pathways to support cell proliferation.

To date, the resulting stratification of results in term of tumour histological or molecular subtype, yielded poor results, allowing only to suppose a relation between invasiveness and intracellular lumen amount, and highlighted the main limit of this study. Indeed, the low frequency of PDO from DCIS and ILC, and from HER2-positive and Basal-like histological and molecular subtype, respectively, was undoubtfully another limitation of this morphological study. However, the low amount of BC-PDOs from ILC, DCIS, HER2+ and Basal-like was simply a result of the epidemiological distribution of these cancer subtypes and should be solved modifying criteria of sample recruitment. Indeed, our study expected them to be recruited samples from consecutive surgery/biopsies without selection by histology or molecular profile. In future, taking into account of this preliminary data, we could design another study, modifying sample size and recruitment criteria in order to perform an ultrastructural analysis of BC-PDOs stratified by histological and molecular subtype.

Finally, electron microscopy has never been used to diagnose breast cancer since all aspects of this disease were well identifiable through histology and immunohistochemistry. However, an in-depth knowledge of the ultrastructural characteristics could also give a valuable contribution in the study of this pathology.

## Conclusion

This study supports the reliability of breast cancer PDO as 3-dimensional model of mammary tissue and identifies some structural features characterizing healthy and cancerous PDO. Besides, it suggests the presence of distinctive traits among different subtypes. Moreover, the ultrastructural characterization of PDO confirms the synergic activity of cells herein included, evidencing the goodness of this model from drug penetration studies.

## Supplementary Information

**Additional file 1:** Dataset PDO.

## Data Availability

Data available in a publicly accessible repository https://doi.org/10.13130/RD_UNIMI/PQCTCX, after publication.
